# Knowledge, attitudes, and practices regarding vitamin D among university students in Quzhou, China: a cross-sectional study

**DOI:** 10.1080/07853890.2026.2665503

**Published:** 2026-05-11

**Authors:** Jian Wang, Yanwen Xu, Weiguo Wang, Cuifang Ma

**Affiliations:** a Department of Gastroenterology, Jiaxing Second Hospital, Jiaxing, Zhejiang, China; b Department of Neurology, The Quzhou Affiliated Hospital of Wenzhou Medical University, Quzhou People’s Hospital, Quzhou, Zhejiang, China; c Department of Infectious Diseases, The Quzhou Affiliated Hospital of Wenzhou Medical University, Quzhou People’s Hospital, Quzhou, Zhejiang, China

**Keywords:** Attitudes, awareness, knowledge, practices, students, vitamin D

## Abstract

**Background:**

Vitamin D deficiency remains common in China, yet recent evidence on university students is limited.

**Methods:**

We conducted a cross-sectional online survey among 397 undergraduates at Quzhou University from November to December 2025 using convenience sampling. A validated questionnaire assessed vitamin D knowledge, attitudes, and practices (KAP) with pre-specified adequacy thresholds (knowledge ≥6/11; attitude ≥3/5; practice ≥4/7). Associations with good practice were examined using logistic regression; item-level subgroup contrasts were explored by sex, attitude, and monthly pocket money.

**Results:**

Awareness of vitamin D, defined as prior recognition of vitamin D, was universal (100%). Only 58.4% (232/397) had adequate knowledge; 66.8% (265/397) had a positive attitude; and 69.5% (276/397) achieved good practice. In multivariable models, women had lower odds of good practice than men (OR 0.57, 95% CI 0.35–0.90); monthly pocket money ≥2000 RMB was associated with higher odds of good practice (OR 3.74, 95% CI 1.70–8.24); and a positive attitude was independently associated with good practice (OR 3.02, 95% CI 1.91–4.79). Knowledge adequacy was not independently associated with practice. Item-level gaps clustered in sun-related behaviors (less daily outdoor walking, <15 min/day exposure, and sun-protection factor use), particularly among women, students with non-positive attitudes, and those with lower pocket money.

**Conclusion:**

Vitamin D KAP was suboptimal in this single-university sample in China. Practice was associated with sex, attitude, and economic means, but not knowledge adequacy alone. Future practice-oriented strategies may prioritize safe sun exposure, affordable supplementation, and on-campus testing for women, economically disadvantaged students, and students with non-positive attitudes.

## GRAPHICAL ABSTRACT

**Figure:**
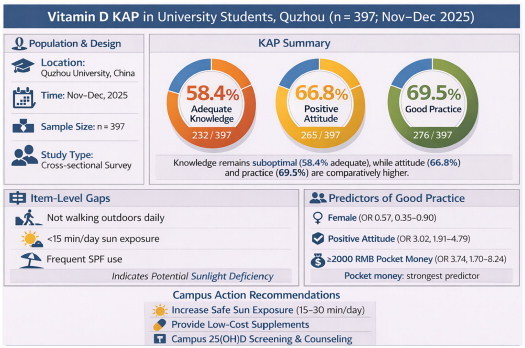

## Introduction

Vitamin D is a fat-soluble prohormone essential for calcium–phosphorus regulation and skeletal health [1,2]. Beyond rickets and osteoporosis, accumulating evidence indicates extra-skeletal associations of vitamin D deficiency (VDD) with autoimmune and infectious diseases, cardiopulmonary conditions, metabolic disorders, malignancy, and all-cause mortality, as summarized in a 2024 expert consensus [3]. Furthermore, the latest 2024 guidelines from the Endocrine Society recommend empirical vitamin D supplementation for individuals aged 1–18 years, those aged 75 years and older, pregnant women, and individuals at high risk of diabetes to mitigate associated health risks [4]. Mechanistic studies suggest that vitamin D exerts immunomodulatory effects by downregulating pro-inflammatory cytokines (TNF-α, IL-6, IL-17) and enhancing anti-inflammatory mediators, lending biological support to these clinical recommendations across chronic disease contexts [5,6].

Globally, VDD affects an estimated 15.7% of the population, with substantial regional heterogeneity [7]. China bears a considerable burden: a meta-analysis of 105 studies reported pooled prevalences of VDD and insufficiency of 20.7% and 63.2%, respectively [8]. University students represent a pivotal group because their lifestyle and health behaviors remain highly modifiable during early adulthood. Notably, a recent international report on university students in developing countries indicated that this demographic lacks sufficient knowledge regarding the health benefits of vitamin D and demonstrates poor attitudes and behaviors associated with it, which is considered a key factor contributing to the high prevalence of VDD [9,10]. However, research on Chinese university students is quite limited. Previous cohort and cross-sectional studies have predominantly focused on children or other specific populations, while evidence specifically targeting university students remains relatively scarce [11,12]. In fact, the most recent study on vitamin D status among university students in China was reported nine years ago, indicating that medical students possess insufficient knowledge about vitamin D, which is associated with unhealthy behaviors [13].

Therefore, we conducted an updated assessment of vitamin D KAP among Chinese university students and examined sociodemographic and behavioral correlates of practice. Our goal was to generate actionable evidence to inform targeted campus health education and low-cost interventions. The graphical abstract summarizes the study design, key findings, and practical implications.

## Materials and methods

### Study design and sample size estimation

A cross-sectional survey design was adopted to assess the knowledge, attitudes, awareness, and practices regarding vitamin D among undergraduate students aged 18 years and above at Quzhou University between November and December 2025. The sample size was estimated using the formula for cross-sectional studies: *n* = (Zα/2)^2^ *p*(1–*p*)/*d*^2^. At a 95% confidence level (Zα/2 = 1.96) with a margin of error (*d*) set at 5%, and considering the variability of KAP reported in previous studies, p was conservatively set to 0.5 to ensure robustness. The calculation yielded a minimum required sample size of 384 participants [14].

### Data collection

A structured questionnaire, adapted from previously published vitamin D KAP instruments, was used to collect information on participants’ sociodemographic and clinical characteristics, together with their KAP related to vitamin D (Appendix 1) [15–17]. Knowledge was defined as factual understanding of vitamin D, attitude as subjective predispositions or preferences toward vitamin D and sun exposure, and practice as actual routine behaviors related to vitamin D acquisition, following established KAP frameworks. The tool was adapted from earlier studies and revised to include validated items for each domain. It comprised four parts: Section A addressed sociodemographic variables such as age, gender, grade level, major, place of origin, parental education, and household income (7 items); Section B assessed knowledge of vitamin D (8 items); Section C evaluated attitudes toward vitamin D and sunlight exposure (5 items); and Section D examined practices concerning vitamin D intake and daily lifestyle behaviors (6 items).

This study was conducted in a single university (Quzhou University), and participants were recruited using a convenience sampling approach. The questionnaire was administered electronically in Chinese through an online link distributed *via* class groups and WeChat. To reduce potential bias, respondents could not move back and forth between sections. Awareness was operationalized as prior recognition of vitamin D and its primary information source, captured using the introductory item at the beginning of Section B.

### Scoring of KAP

In this study, knowledge was defined as factual understanding of vitamin D, attitude as subjective predispositions or preferences toward vitamin D and sun exposure, and practice as actual routine behaviors related to vitamin D acquisition, following established KAP frameworks. The KAP scores were calculated based on participants’ responses. For the knowledge section, each correct response in single-choice questions was awarded one point, while incorrect responses received zero points. In multiple-choice questions, one point was awarded for each correct option selected, and one point was deducted for each incorrect option, until the score for that item reached zero. For the attitude section, a response of ‘Agree’ was scored as one point and other responses as zero, except for the sun-avoidance item (‘I often use a parasol/sunshade and sunscreen lotion to avoid sun exposure’), which was reverse-coded so that Agree = 0, Disagree = 1. For the practice section, the item on daily sun exposure was scored to reflect moderate sunlight exposure as the most favorable behavioral category, rather than assuming that longer exposure was always better. Accordingly, <15 min/day was scored as 0, 15–30 min/day as 1, 30–60 min/day as 2, and >60 min/day as 1. The maximum scores were 11 for knowledge, 5 for attitude, and 7 for practice. Adequacy was defined as follows: knowledge ≥6 points, attitude ≥3 points, and practice ≥4 points; scores below these thresholds were classified as inadequate [16,18].

### Statistical analyses

All analyses were performed using SPSS version 26.0 (IBM Corp., Armonk, NY, USA). Categorical variables were summarized as frequencies and percentages. Associations with good practice were examined using univariate and multivariate logistic regression, and results were reported as ORs with 95% CIs. Forest plots were produced in R (version 4.5.1) with the *forestplot* package. A two-sided *p* < 0.05 was considered statistically significant.

This manuscript was prepared in strict accordance with the Strengthening the Reporting of Observational Studies in Epidemiology (STROBE) guidelines.

### Ethics approval and informed consent

The study protocol was approved by the Ethics Committee of Quzhou People’s Hospital (Approval No. 2025-134). Given the anonymous, minimal-risk online survey design with no collection of identifiable private information, the Ethics Committee waived written (signed) informed consent. Study information was provided in the recruitment notice and on the first page of the questionnaire, and consent was indicated by participants’ voluntary decision to proceed and complete the survey. The study was conducted in accordance with the Declaration of Helsinki.

## Results

### Characteristics of participants

A total of 397 students were included, of whom 56.2% were women. Most participants were 18–23 years old, and the sample spanned all four grade levels. Over half majored in science and engineering, and about 70% reported a rural background. Only 21.4% had a parent with a bachelor’s degree or higher. Monthly pocket money varied across the sample, with 17.2% reporting ≥2000 RMB. Full demographic distributions are presented in Table 1.

**Table 1.** Demographic characteristics of the participants (*n* = 397).

Variables	Frequency (*n* = 397)	Percentage (%)
**Gender**		
Female	223	56.2%
Male	174	43.8%
**Age (years)**		
18–20	237	59.7%
21-23	144	36.3%
≥24	16	4.0%
**Grade level**		
Freshman	218	54.9%
Sophomore	81	20.4%
Junior	70	17.6%
Senior	28	7.1%
**Major/discipline**		
Science and Engineering	226	56.9%
Humanities and Social Sciences	116	29.2%
Others	55	13.9%
**Place of origin**		
Rural	274	69.0%
Urban	123	31.0%
**Highest educational level attained by either parent**		
Below Bachelor’s degree	312	78.6%
Bachelor’s degree or above	85	21.4%
**Monthly pocket money**		
≤1000 RMB	51	12.8%
1000–1500 RMB	137	34.5%
1500–2000 RMB	141	35.5%
≥2000 RMB	68	17.2%

*Note*. Approximate currency conversion for international reference: 1,000 RMB ≈ 145 USD.

### Knowledge responses regarding vitamin D

**Figure 1.**
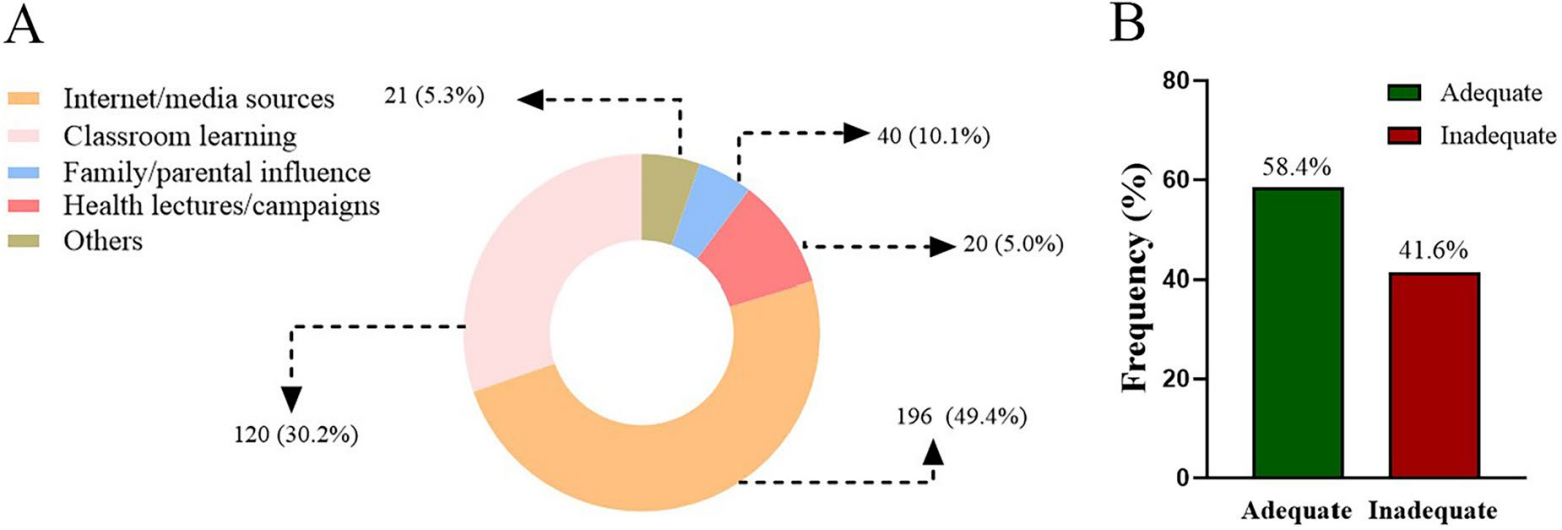
Knowledge responses: (A) primary sources of vitamin D information. (B) Percentage of knowledge response.

As shown in Figure 1A, prior awareness of vitamin D was universal among participants. The main information sources were the internet/media and classroom teaching. Overall, only 58.4% of students had adequate knowledge (Figure 1B). While most respondents recognized the roles of vitamin D in bone health and immunity, knowledge gaps remained for its active form, adequate serum level, and recommended daily intake. Misconceptions about dietary and non-dietary sources were also common (Table 2).

**Table 2.** Knowledge responses regarding vitamin D among study participants.

Variable	Frequency (*n* = 397)	Percentage (%)
**Q1. Vitamin D is good for bone health**		
YES*	392	98.7%
NO	5	1.3%
I don’t know	0	
**Q2. Vitamin D increases the body’s immunity**		
YES*	330	83.1%
NO	53	13.4%
I don’t know	14	3.5%
**Q3. The main active form of Vitamin D in the human body**		
Vitamin D2	95	23.9%
Vitamin D3	72	18.1%
25-hydroxyvitamin D	92	23.2%
1,25-dihydroxyvitamin D*	83	20.9%
I don’t know	55	13.9%
**Q4. Vitamin D belongs to**		
Fat-soluble vitamins*	184	46.3%
Water-soluble vitamins	146	36.8%
I don’t know	67	16.9%
**Q5. The adequate blood level of vitamin D in the body**		
<10 ng/mL	71	17.9%
10–<30 ng/mL	117	29.5%
30–75 ng/mL*	121	30.5%
I don’t know	88	22.2%
**Q6. The recommended daily intake of vitamin D (IU) for adults**		
200	127	32.0%
600*	137	34.5%
800	104	26.2%
I don’t know	29	7.3%
**Q7. The ways to get vitamin d (multiple choice)**		
Diet*	391	98.5%
Sun exposure*	368	92.7%
Exercise	159	40.1%
Air	2	0.5%
Water	5	1.3%
I don’t know	39	9.8%
**Q8. Good dietary sources of vitamin D (multiple choice)**		
Fortified milk*	353	88.9%
Fatty fish (such as tuna and salmon)*	277	69.7%
Vegetables and fruits	187	47.1%
Red meat	166	41.8%
Whole wheat cereal	217	54.6%
Animal liver*	262	66.0%
I don’t know	24	6.0%
Knowledge Score (mean ± *SD*)	397	5.60 ± 1.40

*indicates correct response.

### Attitude responses regarding vitamin D

**Figure 2.**
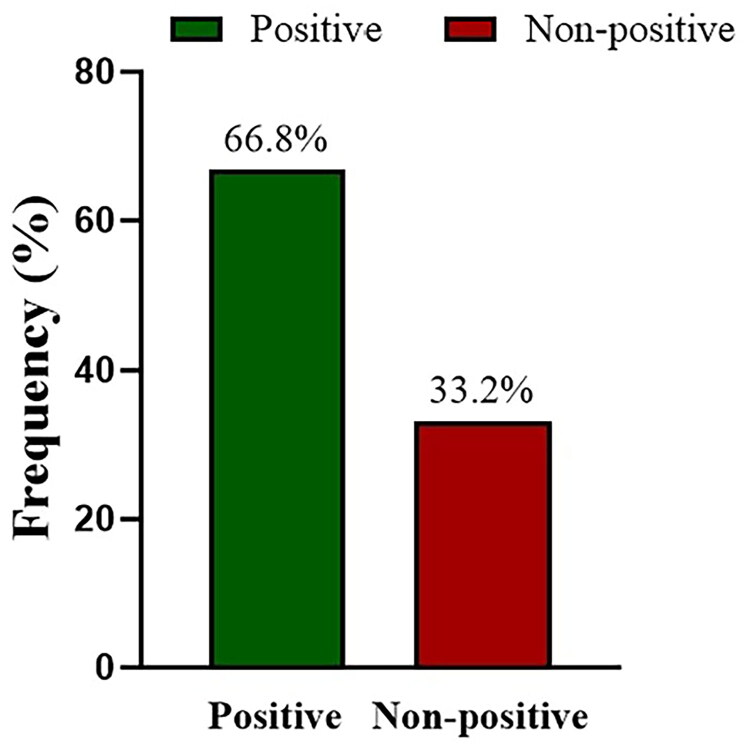
Percentage of Attitude response.

Attitude responses are summarized in Table 3. More than half of the students (217, 54.7%) reported that they enjoyed exposing themselves to sunlight, whereas 79 (19.9%) disagreed and 101(25.4%) remained neutral. About one-third (122, 30.7%) stated that they often used sunshades or sunscreen to avoid sun exposure, while 203 (51.1%) did not, and 72 (18.1%) were neutral. Nearly all participants (372, 93.7%) expressed willingness to undergo vitamin D testing if required by health conditions. Just over half (212, 53.4%) were concerned about having low vitamin D levels, while one-third (132, 33.2%) were neutral. In contrast, only a small proportion (43, 10.8%) expressed willingness to take vitamin D supplements, while 275 (69.3%) disagreed. Based on the scoring system, overall attitudes were two to one: 265 students (66.8%) were categorized as having a positive attitude, while 132 (33.2%) were classified as negative (Figure 2).

**Table 3.** Attitude responses regarding vitamin D among study participants.

Variable	Frequency (*n* =397)	Percentage (%)
**Q1. I like to expose myself to sunlight**		
Agree	217	54.7%
Disagree	79	19.9%
Neutral	101	25.4%
**Q2. I often use a parasol (sunshade or umbrella) and sunscreen lotion to prevent sunshine**		
Agree	122	30.7%
Disagree	203	51.1%
Neutral	72	18.1%
**Q3. I am always willing to undergo test for vitamin D if a medical condition demands it**		
Agree	372	93.7%
Disagree	6	1.5%
Neutral	19	4.8%
**Q4. I am concerned that my current vitamin D levels might be too low.**		
Agree	212	53.4%
Disagree	53	13.4%
Neutral	132	33.2%
**Q5. I am always willing to take vitamin D supplements**		
Agree	43	10.8%
Disagree	275	69.3%
Neutral	79	19.9%
Attitude Score (mean ± *SD*)	397	2.64 ± 0.83

### Practice responses regarding vitamin D among study participants

**Figure 3.**
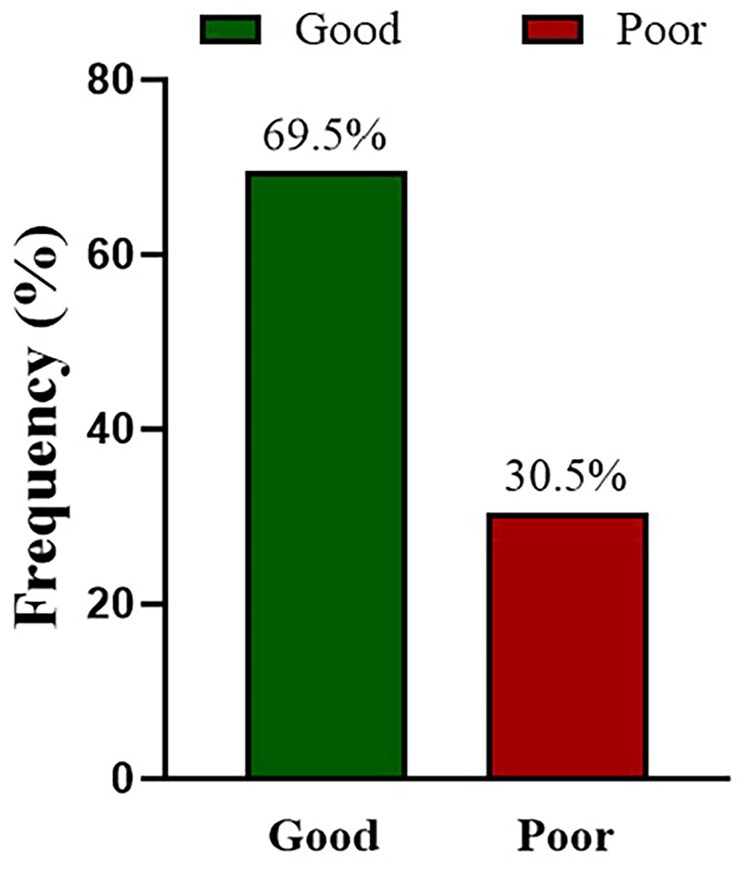
Percentage of practice response.

Practice responses are presented in Table 4. Only a small proportion of students reported ever taking vitamin D supplements (10, 2.5%), and similarly few reported having undergone vitamin D testing (13, 3.3%). By contrast, nearly all participants stated that they purchase foods containing vitamin D (390, 98.2%), and most reported not using sun-protective creams (290, 73.0%). With regard to daily behaviors, 241 students (60.7%) walked outdoors regularly for sunlight exposure, whereas 156 (39.3%) did not. In terms of sun exposure duration, 79 (19.9%) reported less than 15 min, 105 (26.4%) 15–30 min, 159 (40.1%) 30–60 min, and 54 (13.6%) more than one hour per day. Based on the composite scoring, 276 students (69.5%) achieved a score ≥4 and were classified as having good practice, while 121 (30.5%) were classified as poor practice (Figure 3).

**Table 4.** Practice responses regarding vitamin D among study participants.

Variable	Frequency (*n* = 397)	Percentage (%)
**Q1. Ever taken vitamin D supplements**		
YES	10	2.5%
NO	387	97.5%
**Q2. Purchase vitamin D foods**		
YES	390	98.2%
NO	7	1.8%
**Q3. Not using sun protective factor (SPF) contain creams**		
True	290	73.0%
False	107	27.0%
**Q4. Walk outdoor daily for sufficient sunlight exposure**		
YES	241	60.7%
NO	156	39.3%
**Q5. Average length of daily sun exposure**		
< 15 min	79	19.9%
15–30 min	105	26.4%
30–60 min	159	40.1%
> 60 min	54	13.6%
**Q6. Ever undergone vitamin D testing**		
YES	13	3.3%
NO	384	96.7%
Practice Score (mean ± *SD*)	397	3.58 ± 1.21

### Predictors of good practice

**Figure 4.**
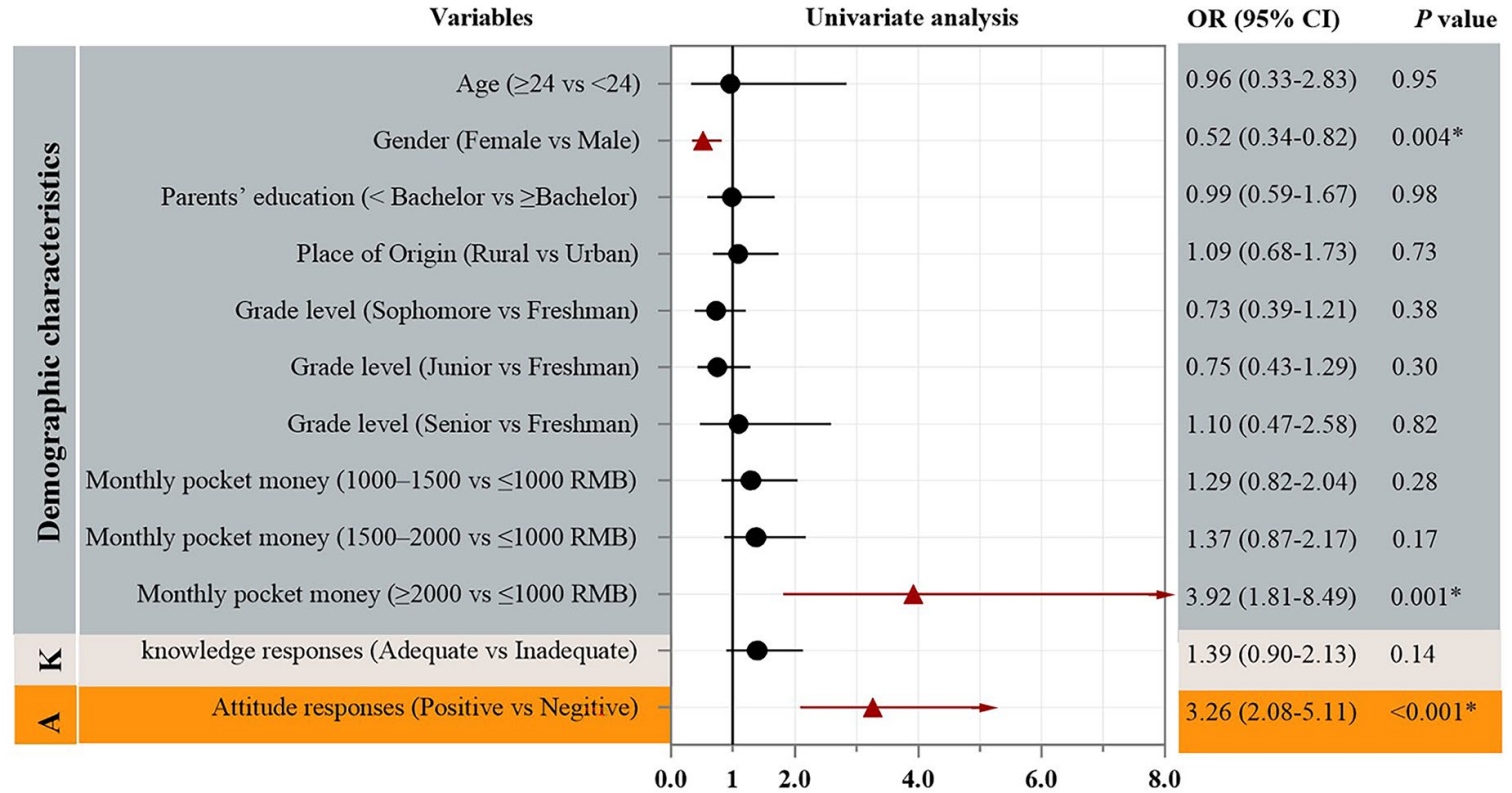
Univariate predictors of practice adequacy.

**Figure 5.**
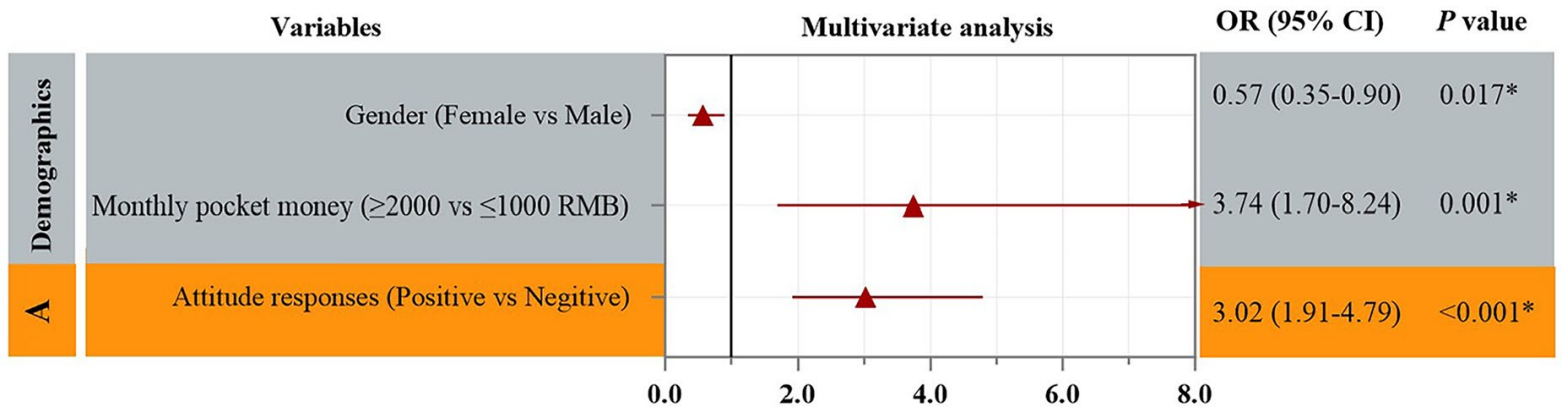
Multivariable predictors of practice adequacy.

In univariate analysis (Figure 4), sex, monthly pocket money, and attitude were significantly associated with practice adequacy, whereas knowledge adequacy and other demographic variables were not. In multivariable analysis (Figure 5), female sex (OR 0.57, 95% CI 0.35–0.90), monthly pocket money ≥2000 RMB (OR 3.74, 95% CI 1.70–8.24), and a positive attitude (OR 3.02, 95% CI 1.91–4.79) remained independently associated with good practice, whereas knowledge adequacy was not.

### Subgroup analysis of vitamin D practices

**Figure 6.**
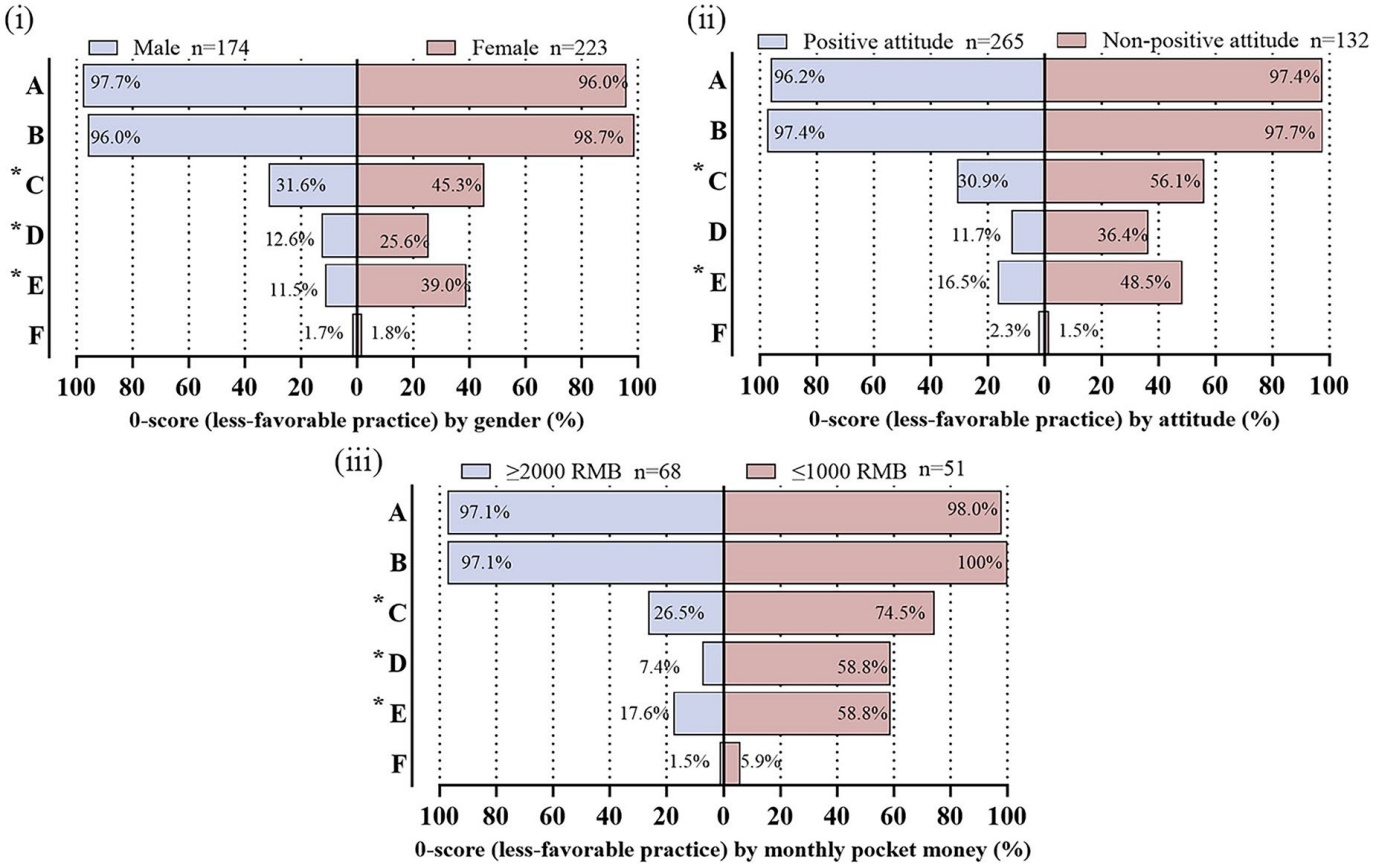
0-score (less-favorable) responses by subgroup: (i) sex (male *n* = 174; female *n* = 223), (ii) attitude (≥3 *n* = 265; <3 *n* = 132), (iii) monthly pocket money (≥2000 RMB *n* = 68; ≤1000 RMB *n* = 51). Items A–F: A, not tested for vitamin D; B, no supplements; C, not walking outdoors daily; D, sun exposure <15 min/day; E, using SPF creams; F, not purchasing vitamin D food. *p* < 0.05 marked with ‘*’.

At the item level, we contrasted the proportion of 0-scores (less-favorable practice) across sex, attitude, and monthly pocket money (Figure 6). The largest gaps were in sun-related items—C (*not walking outdoors daily*), D (*average sun exposure <15 min/day*), and E (*using SPF creams*). Females versus males showed higher 0-scores for C, D, and E (45.3% vs 31.6%, 25.6% vs 12.6%, and 39.0% vs 11.5%; all *p* < 0.05). Students with a non-positive attitude likewise had higher 0-scores for C, D, and E (56.1% vs 30.9%, 36.4% vs 11.7%, and 48.5% vs 16.5%; all *p* < 0.05). Those with ≤1000 RMB/month also exceeded ≥2000 RMB on C, D, and E (74.5% vs 26.5%, 58.8% vs 7.4%, and 58.8% vs 17.6%; *p* < 0.05 for each). In contrast, the proportions of 0-scores for A (*never tested for vitamin D*) and B (*never taken supplements*) were uniformly high across subgroups (≈96–100%), whereas F (*not purchasing vitamin-D–fortified foods*) remained uniformly low (≈1–6%), with no statistically significant subgroup differences for these items.

## Discussion

College students are at a stage where their health behaviors, knowledge, and attitudes are highly malleable, and targeted health education can yield long-term benefits. This study, which reassesses Chinese college students’ KAP regarding vitamin D nine years later, reveals that knowledge and attitudes remain deficient, with notable weaknesses in key practices such as supplementation and sun exposure. Women, individuals with suboptimal attitudes toward vitamin D, and students receiving monthly pocket money of less than 1,000 RMB are more likely to report deficient practices. This pattern may reflect a knowledge–practice discrepancy, where students’ behaviors appear more closely aligned with existing habits and contextual factors than with their level of knowledge.

This cross-sectional survey included 397 university students, all of whom were aware of vitamin D (100%). Media were the primary information source for about half of the participants, followed by classroom learning. Overall awareness levels were higher than those previously reported for populations in Malaysia (90.5%), Egypt (94.3%), and Saudi Arabia (89.6%) [16,19]. This may be attributed to the sample being composed of university students and the high penetration of internet access and health education in China. Conversely, only 58.4% of respondents reported having ‘adequate vitamin D knowledge,’ a lower percentage compared to Nanjing medical students nine years ago (*n* = 350, 68%) and a 2024 study of Saudi medical students (*n* = 291, 86.3%) [13,18]. This disparity may be linked to the fact that no respondents in this study were majoring in medicine. Furthermore, responses to more specialized items, such as ‘appropriate serum 25(OH)D levels’ and ‘recommended daily intake (IU),’ were more dispersed across the options. Although the correct answer had the highest percentage, the advantage was not significant, indicating a lack of understanding of these core concepts. Some respondents may have relied on personal experience or guesswork in their answers. Regarding sources of supplementation, sun exposure and diet are generally recognized; however,40.1% still believe that ‘exercise’ itself can supplement vitamin D, which is significantly higher than the 0.5% reported in the UK and 24.3% in the Arab population [20,21]. Additionally, nearly half mistakenly regard vegetables, fruits, and grains as sources of vitamin D, which is consistent with findings from surveys conducted in Pakistani universities [9]. These findings suggest that university students’ substantive understanding of vitamin D remains insufficient, underscoring the need to strengthen education through coursework and campus media, including greater emphasis on its long-term health implications beyond bone health.

Attitudes among university students towards vitamin D were generally positive, with 66.8% expressing a favorable view. More than half indicated a preference for sun exposure, while approximately half showed no preference for sunscreen. About half of the students expressed concern regarding their vitamin D levels. However, the subjective willingness to take supplements was low (<20%), which is comparable to the 16.3% reported by Ghanaians [16]. In terms of practice, only 2.5% of university students had ever taken vitamin D supplements, a figure similar to the 5–6% rate among students in Nanjing, yet significantly lower than the 26.3% of students at Sultan Qaboos University and 18.5% of American adults [13,22,23]. The proportion of students who had ever taken vitamin D tests was only 1.92%, which is also significantly lower than the approximately 28% of university students in Pakistan [9]. Taken together, the low probability of supplement use, limited vitamin D testing, and insufficient sun exposure indicate that some students may be at risk of unrecognized deficiency. Beyond bone health, such deficiency has been linked to cardiometabolic disturbances, impaired immune regulation, increased susceptibility to infections, and certain chronic diseases, underscoring the need for strengthened preventive education and early intervention.

This study, after analyzing relevant factors such as gender, age, and major, revealed that women are more likely to exhibit inadequate vitamin D practices, primarily characterized by reduced sun exposure and increased sun avoidance behaviors. Similarly, female undergraduate medical students in Sri Lanka demonstrated a heightened risk of limited outdoor activity (adjusted odds ratio ≈ 1.6), while university students in the United Arab Emirates displayed more pronounced sun avoidance behaviors and lower levels of 25(OH)D [24,25]. Furthermore, a cross-sectional study conducted in China from 2016 to 2017 on vitamin D deficiency and insufficiency among children and adolescents found a significantly higher prevalence of insufficiency in girls compared to boys (71.99% vs. 60.42%) [26]. These findings indicate that women, as a demographic group, should be prioritized in vitamin D intervention strategies. Additionally, the study identified that lower monthly pocket money and less favorable attitudes toward vitamin D were associated with poorer practices, which aligns with findings from earlier studies [27,28]. Notably, knowledge adequacy was not associated with better practice in this study. This pattern may reflect a knowledge–practice discrepancy, whereby students’ behaviors appear more closely aligned with their existing habits and contextual conditions than with their level of knowledge. Such a discrepancy should, however, be interpreted as an observed association within cross-sectional data rather than a causal relationship. Similarly, consistent with a KAP survey from Gilgit–Baltistan, Pakistan, that study also found no significant association between knowledge and practice [29].

This study has several limitations. First, the online self-reported questionnaire format may have allowed respondents to base their answers on personal experiences or social expectations, potentially introducing information bias, including social desirability bias. Second, the sample was representative of a single university, and the convenience sampling approach, together with the online format and voluntary participation, may have introduced selection bias, thus necessitating caution when extrapolating the results, particularly when generalizing to university students across China. Third, although our scoring system and adequacy thresholds were adapted from established KAP studies, the use of partial credit and deduction in multiple-choice items may influence how respondents are categorized. Alternative scoring schemes might yield slightly different distributions, and future multi-institution studies could further evaluate the robustness of these classifications. Finally, serum 25-hydroxyvitamin D (25(OH)D) levels were not measured, which prevented a direct examination of the association between knowledge, attitudes, practices, and actual vitamin D status. Future studies are recommended to incorporate objective biological markers to strengthen the evidence.

## Conclusion

Knowledge, attitudes, and practices regarding vitamin D among university students remain suboptimal. Practice adequacy differed by sex, attitude, and economic means: women, students with non-positive attitudes, and those with lower monthly pocket money were less likely to meet practice criteria. In this cross-sectional study, knowledge adequacy alone was not consistently associated with better practice, suggesting a knowledge–practice gap that should be interpreted cautiously rather than causally. Campus strategies may therefore benefit from giving greater attention to these groups and from emphasizing practical supports—such as clear guidance on safe sun exposure, affordable supplementation, and convenient on-campus testing—rather than knowledge-only messaging. Future research involving multiple universities will be essential to verify the robustness and broader applicability of these conclusions.

## Data Availability

The raw data supporting the conclusions of this article will be made available by the authors on request.

## Appendix 1 Study questionnaire

**Participant information and implied consent:**

This anonymous questionnaire is part of a research study on vitamin D knowledge, attitudes, and practices among university students. The study was approved by the Ethics Committee of Quzhou People’s Hospital (Approval No. 2025-134). Because this was an anonymous, minimal-risk online survey and no identifiable private information was collected, the Ethics Committee waived written (signed) informed consent. Participation was voluntary. No names, student numbers, phone numbers, or other direct identifiers were collected. By reading this information and choosing to continue with the questionnaire, participants indicated their consent to take part in the study.

A. Demographics

1. Age in years ___________________

2. Gender:

□ Male

□ Female

3. Place of Origin

□ Rural

□ Urban

□ Prefer not to answer

4. Grade level

□ Freshman

□ Sophomore

□ Junior

□ Senior

5. Major/Discipline

□ Science and Engineering

□ Humanities and Social Sciences

□ Medicine and Health Sciences

□ Others

6. Highest Educational Level Attained by Either Parent

□ Below Bachelor’s degree

□ Bachelor’s degree or above

7. Monthly Pocket Money

□ ≤1000 RMB

□ 1000–1500 RMB

□ 1500–2000 RMB

□ ≥2000 RMB

B. Awareness and Knowledge

How do you know about vitamin D?

□ Internet/media sources

□ Classroom learning

□ Family/parental influence

□ Health lectures/campaigns

□ Others

□ I don’t know

1. Vitamin D is good for bone health

□ YES

□ NO

□ I don’t know

2. Vitamin D increases the body’s immunity

□ YES

□ NO

□ I don’t know

3. The main active form of Vitamin D in the human body

□ Vitamin D2

□ Vitamin D3

□ 25-hydroxyvitamin D

□ 1,25-dihydroxyvitamin D

□ I don’t know

4. Vitamin D belongs to

□ Fat-soluble vitamins

□ Water-soluble vitamins

□ I don’t know

5. The adequate blood level of vitamin D in the body

□ <10 ng/mL

□ 10 ≤ 30 ng/mL

□ 30–75 ng/mL

□ I don’t know

6. The recommended daily intake of vitamin D (IU) for adults

□ 200

□ 600

□ 800

□ I don’t know

7. The Ways to get vitamin D (Multiple choice)

□ Diet

□ Sun exposure

□ Exercise

□ Air

□ Water

□ I don’t know

8. Good dietary sources of vitamin D (Multiple choice)

□ Fortified milk

□ Fatty fish (such as tuna and salmon)

□ Vegetables and fruits

□ Red meat

□ Whole wheat cereal

□ Animal liver

□ I don’t know

C. Attitudes

1. I like to expose myself to sunlight

□ Agree

□ Disagree

□ Neutral

2. I often use a parasol (sunshade or umbrella) and sunscreen lotion to prevent sunshine

□ Agree

□ Disagree

□ Neutral

3. I am always willing to undergo test for vitamin D if a medical condition demands it

□ Agree

□ Disagree

□ Neutral

4. I am concerned that my current vitamin D levels might be too low

□ Agree

□ Disagree

□ Neutral

5. I am always willing to take vitamin D supplements

□ Agree

□ Disagree

□ Neutral

D. Practices

1. Ever taken vitamin D supplements

□ YES

□ NO

2. Purchase vitamin D foods

□ YES

□ NO

3. Not using Sun Protective Factor (SPF) contain creams

□ True

□ False

4. Walk outdoor daily for sufficient sunlight exposure

□ YES

□ NO

5. Average length of daily sun exposure

□ < 15 min

□ 15–30 min

□ 30–60 min

□ > 60 min

6. Ever undergone vitamin D testing

□ YES

□ NO
